# Long-term social assistance recipients’ experiences with an increased monthly payment: a qualitative pilot study

**DOI:** 10.1177/14034948231209369

**Published:** 2023-11-13

**Authors:** Astrid Torbjørnsen, Inger Utne, Borghild Løyland

**Affiliations:** Faculty of Health Sciences, Department of Nursing and Health Promotion, OsloMet – Oslo Metropolitan University, Norway

**Keywords:** Poverty, social assistance, quality of life, qualitative study

## Abstract

**Aims:**

Ten long-term social assistance recipients in a Norwegian municipality received a greater-than-average fixed monthly payment for 12 months. This study aimed to explore whether these recipients with reduced administrative requirements and a fixed monthly payment that was greater than the average social assistance experienced reduced poverty, increased feelings of independence, better daily living, and an improved quality of life.

**Methods:**

The study’s explorative design included 20 qualitative, in-depth, semi-structured interviews and a longitudinal electronic survey for 12 months. The 10 participants had been selected by the local labour and welfare agency based on stringent criteria and are therefore not representative of social assistance recipients in general. Individual interviews were conducted during autumn 2021 and spring 2022. The interview data were analysed using systematic text condensation, and the survey results are presented using descriptive statistics.

**Results:**

The participants included in the project described a reduced experience of poverty. They could buy additional items and set aside money, something they had not been able to do in the past, and meant a great deal to them. They expressed experiencing freedom, gaining a stronger sense of independence, and having lessened feelings of shame. Many of the participants described health issues that were incompatible with working.

**Conclusions:**

**Being given this opportunity led to a feeling of increased dignity and greater inclusion in society among this selected group of participants. They appreciated the simplified conditions and reduced requirements for administrative matters. All expressed that spending 8 months without contact with the social welfare office was a liberation.**

## Introduction

The local labour and welfare agency (NAV) in a municipality in Norway with approximately 50,000 inhabitants wanted to pilot a project with a minimum of 10 persons who had received economic social assistance as their main income for many years. In 2020, the mean social assistance payment for single recipients in this municipality was NOK 13,000 per month. During the pilot project, the participants were to receive a fixed monthly payment of NOK 16,900 for 12 months. The participants did not have to fill in forms, attend meetings, or be under any other control from the local NAV. There were simplified conditions and reduced requirements for administrative matters, such as displaying receipts and submitting invoices linked to payments. The hypothesis was that the individual participants might have a reduced experience of poverty and an increased feeling of independence without having to prove their needs and frequently apply to the local NAV for benefits. The proposal for this pilot project was approved by the City Council on 11 December 2020 (SAK 184/2020).

Economic social assistance is the last safety net in the Norwegian welfare system and provides money for subsistence. It is a means-tested benefit that is intentionally kept marginal to encourage recipients to rapidly become self-sufficient through work. The payment is intended to cover living costs, housing, electricity, consultations with doctors, medicines, and dental treatment. Individuals have a legal right to economic social assistance if they are unable to support themselves, are not entitled to other types of benefits, or do not have someone to support them [1]. The rates of economic social assistance vary depending on the region and the different municipalities. In 2021, 48,666 individuals had received social assistance as their main income for more than 6 of the previous 12 months [2]. Those individuals might be defined as long-term social assistance recipients (LTRs) [3]. Since economic social assistance is intended for short-term use, the existence of LTRs is a social policy problem in Norway. According to the EU’s indicator, the relative poverty line for single individuals in Norway is calculated at NOK 251,600 a year [4].

Equalising social differences, good health, and quality of life are important public health goals in Norway [5], including three UN Sustainable Development Goals. Various studies have shown that LTRs comprise a vulnerable group with a greater risk of poor health status, such as chronic pain, psychological distress, and a weak connection to the labour market [3,6–9]. Many have experienced economic stress in their childhood home, long-term bullying, different types of abuse, loneliness, and dropping out of school before the age of 16 [10]. A study of social assistance dynamics trajectories following young recipients between 18 and 24 years old across a span of 20 years found one trajectory that was distinguished by frequent shifts between unemployment, social assistance, and some low-paid work [11]. This trajectory was named ‘long-term unemployment and health issues’ and covered 10.8% of first-time social assistance recipients. The authors used sequence analyses to explore and map the trajectories of longitudinal administrative data collected and linked by Statistics Norway. Another study confirmed that poor health and being on social assistance for the long term is an issue [9]. This study connected longitudinal administrative data with a survey of LTRs for the period 2005–2013. After 9 years, the findings showed that the main source of income for 22% of these persons was still social assistance.

The World Health Organization defines quality of life as an individual’s perception of their position in life in the context of the culture and value systems in which they live and in relation to their goals, expectations, standards, and concern [12]. On average, the quality of life is high in Norway, but it is unevenly distributed, and some groups experience a poor quality of life [13]. In a previous study, LTRs rated the physical and mental components of their health-related quality of life as lower than in the general Norwegian population [14]. In the present study, we wanted to know about the LTRs’ subjective experiences of being part of this pilot project.

Therefore, this study aimed to explore whether 10 LTRs experienced reduced poverty, increased feelings of independence, better daily living, and an improved quality of life when their administrative requirements were reduced and they were given a fixed monthly payment that was higher than the average social assistance.

## Methods

### Design

This study’s explorative design included qualitative, in-depth, semi-structured interviews and a longitudinal electronic survey for 12 months. Some administrative information from the local NAV was also included. The present study was registered by the Norwegian Centre for Research Data (ref. no. 490088). The project was originally planned to last 12 months each participant being interviewed three times. The first was undertaken at project commencement, the second midway through the pilot and the third was scheduled to take place after 12 months when the project had ended.

### Participants and sampling

The local NAV invited 10 LTRs to participate in this pilot project, and all accepted. The inclusion criteria were having received social assistance for several years, not being obliged to participate in active labour market programmes, and not being covered by other benefits. They had to be single, not supporting children, able to dispose of their own payment, and able to speak the Norwegian language. Exclusion criteria included complex problems related to substance abuse and psychiatry, because such issues require a more complex and comprehensive follow-up. The 10 participants had been selected by the local NAV based on stringent criteria. The researchers had no influence over the selection of project participants, yet managed to successfully conduct two rounds of interviews with all participants included in the project.

In May 2021, the local NAV asked Oslo Metropolitan University (OsloMet) to evaluate the pilot project. We had four collaborative meetings with NAV and collaborated on information and development of the project’s interview guide and questionnaire. Furthermore, we discussed other information we needed. The collaboration took place digitally and we shared the documents in planning. NAV was not involved in the interviews or in the analyses and writing, nor did they follow up with the participants during the period. The interview guide was discussed with the first two participants interviewed. The research team (the authors) has extensive experience with research into the quality of life among people with various illnesses and health challenges, including long-term recipients of social assistance. All the participants signed a written informed consent form after being informed about the study. The forms are stored in a safe at OsloMet, and only the pilot project leader has access to them.

### Data collection procedures and setting

The researchers developed a semi-structured interview consisting of eight open-ended questions to ensure rich descriptions of the participants’ life, wishes and relations with NAV. The interview questions were formulated using everyday expressions and language that were adapted to the topics of interest (Appendix I, Supplemental material). Additional probing questions were added during the interviews to enrich the data. The first and last authors met the participants for the first interview 1 to 3 months after receiving their first fixed monthly payment in August/September 2021 and the second from January to April 2022. The last author led all the interviews with the participants because she has extensive experience undertaking qualitative interviews and knows the research field and the various challenges this partly marginalised group has. This approach ensured consistency and reliability as two interviewers reduce researcher bias in the questioning of the participants. The first author also asked the follow-up questions.

Most of the interviews took place at a public library in the centre of the participant’s home city, and the library offered free, suitable group rooms in a neutral place without disturbances. However, the first interview was conducted at the home of one of the participants, the second at NAV, two participants did not want to meet physically, and four interviews were conducted by telephone. The interviews lasted about 1 h, ranging from 25 to 70 min, and were tape-recorded and later transcribed verbatim by the last author. The Service for Sensitive Data, which is designed for storing and post-processing sensitive data, was used to collect and store the qualitative and quantitative data [15]. The self-report survey was sent to the participants on a monthly basis for 1 year through Nettskjema, a secure tool for managing online data collection on all platforms [16]. The survey comprised nine questions about various aspects of quality of life, such as general health, satisfaction with life, symptoms of anxiety and depression, and a question about loneliness (described in Appendix I, Supplemental material).

### Data analysis

The analytic approach was adapted from systematic text condensation, a strategy of analysis in four steps developed from traditions shared by most methods for qualitative data analysis and described by Malterud [17]. In the first step, the first and last authors read all the interviews (a total of 284 pages) to get an overview of the data and to grasp a sense of the participants’ experiences during the pilot project and to repeatedly discuss the core messages. In the second phase, they identified meaning units focusing on the participants’ life experiences in the pilot project period. After some weeks, the second author read and individually coded the transcriptions, the two other authors reread the interviews, and all authors discussed the emerging categories first written down and discussed by the first and last authors. In the third phase, the authors reduced and identified new categories and subcategories. The first and last authors ensured validity by again checking the original transcripts, and all three authors discussed and agreed on the categories each had found by reading all interviews. In the fourth phase, categories were synthesised, developed into main and subcategories, and illustrated in the findings. The first author suggested a text that the second and third authors approved. Quotes showing the categories were translated following decontextualisation, coding, synthesis, and recontextualisation. The final thematic categories, subthemes and exemplar data were documented and discussed with all three authors. Examples of the steps taken in our evaluation of trustworthiness, credibility, dependability, and transferability are shown in Table I [18,19]. The software programme NVivo was used to analyse the in-depth interviews.

**Table I.** Methods used to assess trustworthiness, credibility, dependability, and transferability.

Step	Techniques	Examples of techniques used
Trustworthiness	Precise and consistent data analysis	• Two researchers were present at the interviews
		• All three authors read and separately coded the interviews – multiple observers
		• All three researchers discussed coding and themes
		• Rewriting of themes and repeated consensus between the researchers • Maintained a reflexive approach throughout the project
Credibility and dependability	Correct and accurate findings Data triangulation	• Read and reread the interviews
		• Review codes and themes interchangeably
		• Interpretations and conclusions supported by the data
		• Data triangulation, quantitative data supporting qualitative findings
Transferability	Findings applicable to other settings	• Describing the setting in detail

## Findings

### Information from the local labour and welfare authority

This municipality’s mean economic social assistance increased to NOK 14,000 monthly for 2021, and the 10 participants received NOK 19,500 monthly. Their ages ranged from late 30s to early 60s, and they had, on average, received social assistance as their main income for 12.2 years in the period 2000 to 2021. In May 2022, the City Council extended the fixed monthly income period from 12 to 18 months. The reason for the extension was that the City Council wanted to know how those included had experienced being part of the pilot project. Before this point, none of the participants had contacted NAV, asked for extra money, or contacted NAV about unpaid bills for electricity or housing. The researchers have therefore postponed the final participant interviews, which had been planned for the end of the 12-month pilot.

### Findings from the analyses of the in-depth interviews

Through the analyses, we identified three main categories: ‘A sense of relief and a feeling of reduced poverty’, ‘Minor changes in daily living’, and ‘Uncertainty and concern for the future’.

### A sense of relief and a feeling of reduced poverty

In both interviews, the participants expressed a sense of relief from the pressure of having to reveal the details of their finances and spending. Having been in the system for many years and frequently having to prove their need for financial support by giving the authorities oversight of their bank accounts was described as a considerable burden. It caused feelings of worthlessness.

In my head, I am thinking, ‘Yes, now I have universal income’. In my head, that is how I think about it. And then . . . It feels a bit more dignified, really (Interview (Int.) 9).

When the interviewees were asked about what being in this pilot project had done for them, they expressed experiencing greater freedom from having more disposable income. They had gained a strong sense of independence. In addition, no authorities were breathing down their necks wanting to access details of their actions. This opened up the possibility of buying what they needed. Furthermore, they had predictability in their finances because the money arrived on a fixed date or on two selected dates. They were able to manage their bills, such as rent and electricity. They did not have to justify their expenses to anyone. They said they had money all month, and, therefore, much less worry about not having money and less anxiety about being unable to pay their bills on time. Their confidence only improved while being in the pilot project. When the interviewer asked whether participation in this pilot project had affected how they lived their lives, one of the participants stated,

Yes, absolutely. It is weird that the somehow the combination of the sum of money, paying bills and rent, at least for my part, makes me forget that I am in the lowest category when it comes to social ranking (Int. 18).

It had been a relief not having to go ‘down there’ to the social welfare office and feel subjugated, as well as being able to decide for themselves how to use the money.

Yes, so you start feeling physically ill because it is like you have to go there and ‘beg’ every time (Int. 19).

Not having to give anyone access to their bank account has also been perceived as raising living standards and subsequently inspiring confidence. Moreover, spending 8 months without having to have contact with the social welfare office was a liberation: there had been no need to even enter the building. This had increased self-esteem, self-confidence and decreased the participants’ sense of loneliness. Some had used the opportunity to pay debts or repair their cars. Not being ‘controlled’ had been a relief for everyone. It had made a difference in many ways: less shame, the possibility to get out from under the authorities’ purview, feeling encouraged, and not having to rely on borrowed money.

It was like a weight off my shoulders. Not dealing with the shame every 3 months of applying for new [social assistance]. So now I feel nice in the way I do not have to do that . . . it has cheered me up quite a lot (Int. 15).

Many interviewees talked about the relief from embarrassment or shame having been given control of their own finances. Over the years of receiving economic social assistance, they had had to demonstrate their need for support by providing the authorities with complete oversight of their finances and bank statements. Participants reported feeling humiliated during these stressful inspections. Furthermore, all additional income, such as gifts from family and friends, reduced their economic social assistance.

To put it quite frankly, you are no longer required to be incapacitated. When you need to have an audit of everything you have in your own life. It is just like you are under the age of 18, and your parents have a say over you. That is exactly how you feel. Essentially incapacitated (Int. 1).

They expressed considerable relief at being allowed to manage their income with complete freedom.

That you cannot escape. They are lingering above you the whole time. You do not have the freedom you should have. I feel much freer now, in that sense (Int. 8).

They were deeply thankful for the opportunity. Being dependent on the system was described as devastating. One participant depicted it as going around in a circle,

You will be stuck in this system. It is like that, you keep going around in circles, and then there are periods when you get quite a bit ‘down’. It takes a mental toll . . . (Int. 8).

One of the participants asked questions about the backgrounds of the case managers. All of them said that they changed case managers often. They attended mandatory courses that they felt were unnecessary, where they had to pretend they were interested. For example, attending a course on writing a curriculum vitae reportedly gave them nothing. It was experienced as very unpleasant that participants had to provide access to private matters and relinquish control. Some said that no one else should have anything to do with how they use their money.

### Minor changes in daily living

Life for the participants mainly stayed the same; they described small changes in their daily life, but did most of the things they had done before.

No, it changes nothing, the only thing which it changes is that . . . my personal life is taken care of by myself (Int. 4).

I thought initially that it would become better, that I would get more money to have more to play with. But it is not. But it is a good thing that I can pay rent and electricity when that comes (Int. 21).

Everyday life consisted of being at home, watching the television, listening to music, going out walking, hobbies not involving high costs, and visiting or interacting with family or a few friends:

Monotonous. Very repetitive. It is like yesterday could as well have been the day today and that today could have been tomorrow. There are very few changes in things happening (Int. 10).

In total, several of the participants spent their days mostly at home. One of them suspected that this was the norm for others, too.

I spend a lot of time at home, hm. I bet it is normal for many in my situation (Int. 3).

When we asked another participant if the days were long, he replied,

I get very tired. I need to sleep in the daytime, and sometimes I fall asleep on the sofa. Exhausted from all the thoughts (Int. 20).

They expressed an optimistic hope of having sufficient money for what they felt they needed during a month. They treasured being able to use the extra money for small but valuable items like new shoes, clothes, makeup, food, Christmas and birthday gifts, a needed freezer, tobacco, curtains to keep warm, fees for getting back a driver’s licence, paying the dentist, taking animals to the veterinarian, saving money, paying off private debts, or taking a short trip. The small items were not defined as basic needs but were essential to them. Life had not changed that much, but it helped to have money in their accounts, and the change in feeling was described as the diminishing sense of being lowest on the social ladder. They felt more like ‘normal’ people. They described the feeling of becoming like everyone else or of becoming a better person. However, despite more money at their disposal, they still lived below the poverty line.

Some participants described loneliness as a part of their everyday lives.

Yes, it is a lot of the same stuff over and over again. Yes, but like I said, I do not have any particular problems with being alone. So it is all right. . . . So, I am never out on the town alone, in a pub or a restaurant or anything like that, so. . . So, in that respect, it can be quite lonely (Int. 3).

Well, I have a dog. Yes, I can go on walks with it, but yes, apart from that, I am alone quite a lot. A lot of time alone (Int. 21).

### Uncertainty and concern for the future

The opportunity to participate in the pilot project was a unique experience for the chosen persons. They described how having to return to receiving social support as before would be a challenging transition and a would feel like a big step back. They felt lucky to have been given this year free from the constant pressure, of looking for ways to support themselves without finding a way out of the situation. However, the prospect of a limited time remaining on the higher level of support left them constantly worried about the future.

The only thing that is a shame about this pilot project is that it is over in about half a year, or 3 or 4 months, or something like that, I believe. And then, I will be back on the usual social assistance. I find it quite awful . . . It is like regularly receiving cakes, but now I have to go back to dry slices of bread (Int. 22).

Some participants hoped to clarify their situation, asserting that they could not work for a living for health reasons. They were not in the same position and did not have the same hopes as when they were younger. They were mentally preparing for a change for the worse. As one of the participants reflected in the second interview:

So, in that sense, it is better. Even though I am paying for electricity and all of that stuff myself there is quite a bit more money compared to those NOK 6,550 you receive in July or August, if you receive any at all. The time passes really quickly, and then, all of a sudden, you are there (Int. 16).

I am obviously very excited because this is a trial lasting for a year. And then, when the year passes, I need to start applying again. I do not know how it will become, and I do not know how to feel, to be quite honest (Int. 6).

They felt that they did not have access to the labour market. They previously had the strength to stand in work situations, a strength they did not possess anymore. They described a disease burden that was perceived as incompatible with being at work. In addition to this, older age and a lack of education were further barriers to accessing the labour market.

And it is probably more difficult for me to get a job compared to someone younger with an education. Because I have no education. I only have 9 years of elementary schooling. And you need papers regardless of what job you want. I do have a truck licence, however (Int. 10).

Had I been 25 now, I would not be on alternative forms of welfare, but I am 58 (Int. 5).

Health issues that make it impossible to work must be documented sufficiently to gain financial benefits beyond social assistance. However, this seemed complicated, and individuals could fall outside the welfare system without being able or having the health to address these challenges.

It should probably not be for everyone, but at least for those who have been on welfare for a long time and do not get anywhere. Who are stuck in the system. In that case, I think it could go well. It makes my life more dignified. It is not very dignified to be on social assistance (Int. 22).

One of the participants stated that being in a situation that was not clarified was the worst,

Uncertainty, that is probably the worst problem when you do not know (Int. 17).

Quality of life issues were important. One of the participants described how experiencing oneself as a participant in society and feeling well oneself were closely linked to quality of life.

Well, it is like I said about making everyday life easier, and now I am at least 100 percent sure that I have money throughout the whole of the month. It means everything. It is number one, really. If your quality of life is not good, you cannot do anything to improve the lives of people around you (Int. 10).

The incentive to start working was lacking for some participants as they had debts, and their potential income would be reduced, as the authorities would garnish their wages.

It is not exactly motivating because they subtract from my salary. I do not owe detrimental amounts of money, but it is at least a couple of NOK 100,000 at least (Int. 2).

One of the participants reflected on the possibility of making changes and getting a better life while being poor.

It is quite absurd to then think poverty and problems are incentives to get a job and make changes in your life. It is completely absurd (Int. 3).

### Quantitative measures

**Figure 1.**
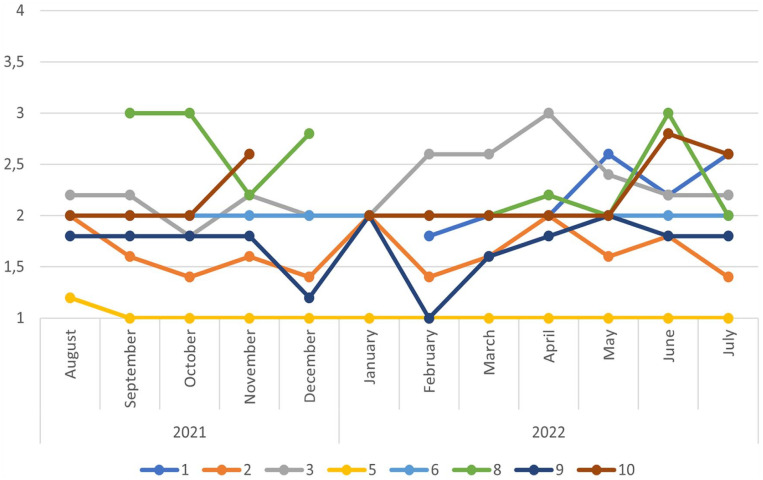
The Hopkins checklist (SCL-5) sum score ranges from 1 (‘Not at all’) to 4 (‘Extremely’), with a cutoff at 2.0. Higher scores indicate the presence of symptoms of anxiety and depression.

**Figure 2.**
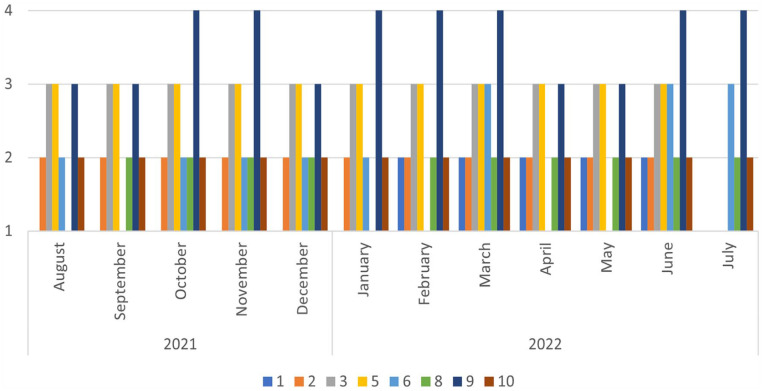
Loneliness. The scale ranges from 1 ‘Often’ to 4 ‘Never’.

According to the quantitative registrations during the year, 8 of the 10 participants responded to the questionnaire every month from August 2021 to July 2022. The results of the Hopkins checklist (Figure 1) showed that symptoms of depression and anxiety were stable throughout the year. Regarding loneliness (Figure 2), the participants mainly reported sometimes being lonely. The total scores for general health (Appendix II) ranged from 1 to 5, where only one of the participants scored 4 or above for more than 1 month during the year. In response to the satisfaction with the life question (Appendix III), the range was from 3 to 9, with slight individual variations, mostly greater than 5.

## Discussion

This pilot study aimed to explore whether 10 long-term recipients of social assistance experienced reduced poverty, increased feelings of independence, better daily living, and an improved quality of life by receiving a fixed monthly payment higher than the usual economic social assistance. The findings from this study showed that the participants expressed a great sense of relief and a feeling of enhanced worthiness while participating in this pilot project. However, this occurred even though the participants experienced only minor changes in their everyday lives. The ability to make small changes was valuable.

### Poverty

The individual participants described a reduced experience of poverty during the first 8 months of the pilot project. They reported how they were able to buy other items and set aside money for future expenses, something they had not been able to do in the past but was of great importance to them. This is not surprising, because their payment increased by an average of 39% from the original, and they were used to living on a significantly smaller monthly amount.

They stated that the predictability of the fixed monthly payments gave them vital assurance. They knew exactly how much money they would be getting and when they would receive it. They could pay their living expenses and electricity on a fixed day. They also had one less administrative chore in managing their expenses, as they had previously been required to deliver their utility bills to NAV for actioning. They described the stark contrast between the current conditions and the usual dependence on NAV, with demands for applications, receipts, and access to bank accounts showing how they spent their money. They had been given time out from these aspects of life, a break from the dependence on caseworkers, something they deeply appreciated.

On average, these individuals had received social assistance as their primary income for 12.5 of the past 20 years. They described being on NAV as an addiction and feeling that they would never get out of the system.

Even though the participants all felt they had received a significant increase in income, they were all still below the poverty line, as described by the EU. Throughout 2022, electricity prices increased in addition to expenditure on food. The government anticipated this would lead to a challenging situation for the least advantaged, and social assistance recipients therefore received extra financial support from early on. Due to this compensation, the increased electricity prices did not emerge as a topic during the interviews to any great extent. Statistics show that the number of low-income households in Norway has decreased over the last years from 2021 onwards [4].

### Health issues

Statistics suggest that problems can accumulate in particular groups in society. People receiving long-term social assistance are most often subjected to three or more welfare issues/problems. The most common combinations are decreased health and being unemployed [20].

This study’s findings showed that the participants’ health situations were complex. They talked about physical and mental illness, but these issues were still not sufficiently documented for them to qualify for more permanent social benefits. Previous research has documented that accessing paid labour is much more difficult for people with health problems, especially those with mental health issues [7, 21]. A study found that better mental health is a predictor of a decrease in recipients’ financial hardship [22].

In addition, findings from the quantitative survey showed that many of the LTRs reported high scores for symptoms of anxiety and depression. They would probably have been diagnosed as having such symptoms if they had had sufficient contact with health services [23]. This aligns with a previous study of a large Norwegian cohort [14]. Much of the uncertainty about the future is related to these matters. The LTRs feared that they would not have the opportunity to become self-supporting or were uncertain whether they would qualify for other support schemes. Research shows that this uncertainty is not unfounded [7,21,24]. Clarifying the state of health of social assistance recipients is a complex procedure for NAV [25]. NAV aims to provide close and individual follow-up with this group, which is necessary but can prove challenging [25]. If the person also has mental health challenges, this can be so demanding that it is given lower priority [26]. The lack of priority seemed to have an impact on the participants’ daily life, and their feeling of shame.

Further, while we need to be cautious when drawing conclusions based on the quantitative data, it appeared that, despite a reduction in poverty, symptoms of depression, anxiety and loneliness remained stable through the year. This suggests that increased governmental financial support may not necessarily ease challenges related to mental health and loneliness for this particular group. The psychological impact of poverty may have long-lasting effects even if financial circumstances are improved. Any attempts to improve quality of life could demand a more nuanced understanding.

### Loneliness

The participants in this study lived different lives. Nevertheless, what they had in common was that they were not very active outdoors and had few social networks. One might argue that this would not change even if they had somewhat better finances. This aspect was also reflected in the current study. In the interviews, several participants expressed that they were often alone and often felt lonely. They could deal with being lonely, well aware that others are lonely as well. Previous studies have found that loneliness characterises LTRs [7]. Many also live hazardous lifestyles (e.g., drug misuse) and risk dying much earlier than the rest of the population [27].

In addition, persons living alone have an increased risk of becoming LTRs [24]; however, this was an inclusion criterion in the present study. There could be other possible explanations or barriers as to why their lives seem to remain unchanged in this respect, even when they have increased financial assistance. A plausible explanation is their state of health. Hazardous lifestyles appeared to be a particular risk among males: some described the use of drugs, close friends who were dead, and family conflicts.

### A selected sample

A source of uncertainty in our results was that the group of LTRs were carefully selected by the welfare authorities based on several criteria to ensure that those given this opportunity could handle the financial responsibility while participating in this pilot project. Although the interviews revealed that the individuals given this opportunity were markedly different from one another, they nevertheless had the shared experience of having been in receipt of economic social assistance for a long time from a system designed only to be a short-term solution.

It is also possible that if the pilot project inclusion criteria had been less stringent and more people had been given this opportunity, we would have seen different outcomes. We do not have statistics on how many people in this municipality would qualify for this pilot project. A Norwegian registry-based study that examined predictors of why people became long-term social recipients [28] could not find any clear indicators of the need for long-term assistance. However, the authors discussed the possibility of coincidence, such as the importance of successful follow-up from a caseworker in NAV or a generous workplace, which the registry study could not capture [28]. The participants in the present study had frequent changes of caseworker and described this mainly as a burden, not a support. The Norwegian Board of Health Supervision reports that accessibility to the services in NAV has largely been digitalised, meaning there are now fewer touchpoints. Even if the level of accessibility is suitable for many, it can be challenging for some [29]. Such perspectives may be of significance for quality of life. And even if the participants are assured by receiving a slightly higher income, there is no guarantee that this will lead to any substantial change in improved daily living across every aspect of life.

### About the transfer value of the study – further research

There are still many unanswered questions about the complexity of the situations of people who receive long-term social services and remain unable to gain access to the labour market or qualify for more permanent governmental support schemes. A recent scoping review investigating studies dealing with health inequalities discusses the need for more research on health challenges and access to the labour market. It also points out that more qualitative studies are needed [30]. A recent rapid review of health and well-being inequalities in Norway particularly mentions LTRs as a group who are living below the poverty line and as vulnerable [31]. Further research should be undertaken for a more in-depth investigation of the resources and needs of LTRs to develop innovative and sustainable solutions for this group. The 10 participants were not representative of social assistance recipients in general. In reference to the strict inclusion criteria described above, transferability can be discussed to the extent that others are chosen with the same criteria. The inclusion criteria to speak Norwegian and have documented long-term residence in Norway were, according to the local NAV office, designed so that the participants could share their reflections with the researchers without communication problems. Future projects will therefore not necessarily have this as a requirement. Further, the persons included had lived for many years on social assistance and would have difficulty finding a job. Unfortunately, this is not a unique situation [32].

### Limitations

It is essential to note the possible bias in the quantitative responses, as this study is reporting on a small sample of respondents. The data were collected exclusively during the pilot project period and were not checked against the respondents’ data before the pilot project started or with any control group of LTRs who were not been part of this pilot project. We know that the participants did not contact NAV within the period for support. The fact that we only have survey data from eight participants provided a minimal opportunity to interpret the relevance of the data. Therefore, we have only given their responses descriptively to illustrate the variation throughout the year, and the results must be interpreted cautiously. Further, the researchers’ prejudgments may have affected the interview and the analysis in several ways. However, Table I lists the methods used to ensure the reliability, credibility, dependability and transferability of the findings. The participants may have expressed positive experiences because they had been granted a financial boost. Through the data collection, analysis and interpretation of results, we maintained a reflective approach. Our questions were open, to facilitate rich and nuanced responses, and we believe the participants felt comfortable enough to share their honest opinions.

## Conclusions

This pilot study showed that life became better when the participants had more money, and they appreciated not being obligated to meet case workers, who changed frequently, which therefore made it more difficult for the recipients (i.e., they repeatedly had to explain who they were and why they were there to new case workers). Furthermore, they experienced being less poor and feeling less shame, were more independent, and had a greater sense of independence and freedom. According to WHO’s definition, the participants quality of life had increased, something several of them explicitly referred to during the interviews. Everyone expressed that it was liberating not to have to give NAV access to their bank accounts. However, the essential elements of their lives had not changed. If we are to equalise social inequality in health, we need to start addressing this among LTRs.

This was a small study with only 10 participants selected to pilot this measure. To identify whether this could be a feasible solution to ensuring that long-term recipients of social assistance have a better life, it will have to be tested on a larger scale. This will consequently require the political to spend money to reduce poverty.

## Supplemental Material

sj-docx-1-sjp-10.1177_14034948231209369 – Supplemental material for Long-term social assistance recipients’ experiences with an increased monthly payment: a qualitative pilot study Supplemental material, sj-docx-1-sjp-10.1177_14034948231209369 for Long-term social assistance recipients’ experiences with an increased monthly payment: a qualitative pilot study by Astrid Torbjørnsen, Inger Utne and Borghild Løyland in Scandinavian Journal of Public Health

sj-png-2-jmo-10.10.1177_14034948231209369 – Supplemental material for Long-term social assistance recipients’ experiences with an increased monthly payment: a qualitative pilot study Supplemental material, sj-png-2-jmo-10.10.1177_14034948231209369 for Long-term social assistance recipients’ experiences with an increased monthly payment: a qualitative pilot study by Astrid Torbjørnsen, Inger Utne and Borghild Løyland in Scandinavian Journal of Public Health

sj-png-3-jmo-10.10.1177_14034948231209369 – Supplemental material for Long-term social assistance recipients’ experiences with an increased monthly payment: a qualitative pilot study Supplemental material, sj-png-3-jmo-10.10.1177_14034948231209369 for Long-term social assistance recipients’ experiences with an increased monthly payment: a qualitative pilot study by Astrid Torbjørnsen, Inger Utne and Borghild Løyland in Scandinavian Journal of Public Health
