# Supplementary material for: Long-term social assistance recipients’ experiences with an increased monthly payment: a qualitative pilot study
Source: Scand J Public Health. 2023 Nov 13;52(8):907–17. doi: 10.1177/14034948231209369 (PMC11626837; doi:10.1177/14034948231209369)
Supplement: sj-docx-1-sjp-10.1177_14034948231209369 – Supplemental material for Long-term social assistance recipients’ experiences with an increased monthly payment: a qualitative pilot study [file sj-docx-1-sjp-10.1177_14034948231209369.docx]

Appendix I. Interview guide

| **Questions** |
| --- |
| - What are you up to during the day? (being alone, activities) - If you wanted to describe your day, what would you say? - Has this had any impact on how you live your life? - Has this made life change in any way? - Do you enjoy life? - This contact with the people at NAV, what is it like? - If you were to want something, is there anything you could want in life? - Is there anything you think we should know that we have not asked? |
