# Supplementary figures and images for: Long-term social assistance recipients’ experiences with an increased monthly payment: a qualitative pilot study
Source: Scand J Public Health. 2023 Nov 13;52(8):907–17. doi: 10.1177/14034948231209369 (PMC11626837; doi:10.1177/14034948231209369)

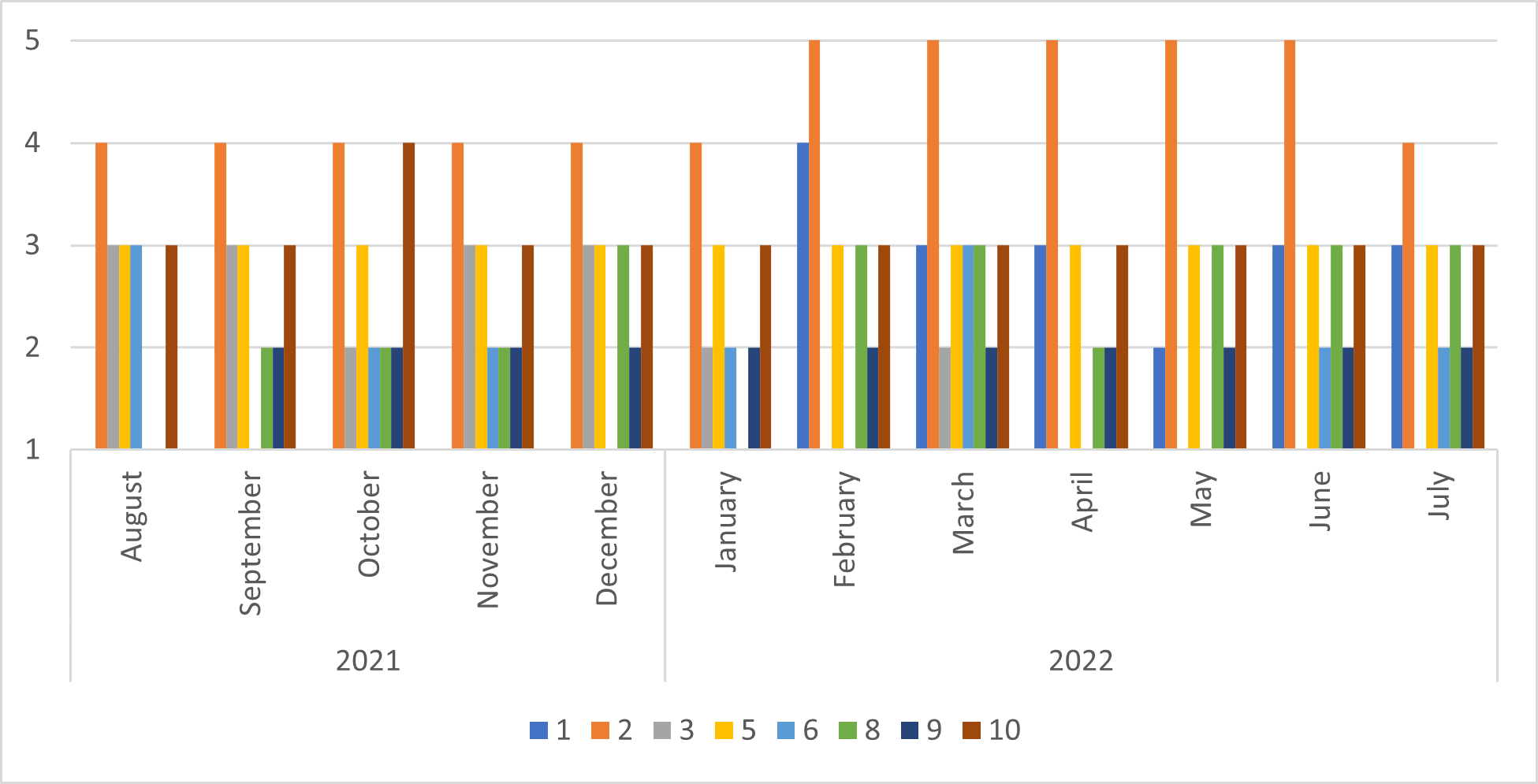

Supplement: sj-png-2-jmo-10.10.1177_14034948231209369 – Supplemental material for Long-term social assistance recipients’ experiences with an increased monthly payment: a qualitative pilot study [file sj-png-2-jmo-10.10.1177_14034948231209369.png]

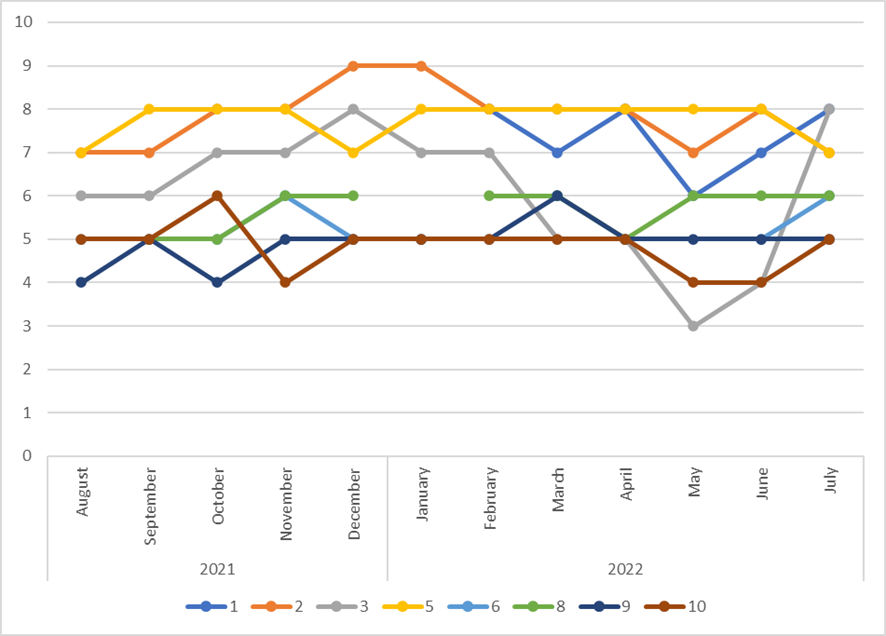

Supplement: sj-png-3-jmo-10.10.1177_14034948231209369 – Supplemental material for Long-term social assistance recipients’ experiences with an increased monthly payment: a qualitative pilot study [file sj-png-3-jmo-10.10.1177_14034948231209369.png]
